# Metabolomics-Based Clinical Efficacy and Effect on the Endogenous Metabolites of Tangzhiqing Tablet, a Chinese Patent Medicine for Type 2 Diabetes Mellitus with Hypertriglyceridemia

**DOI:** 10.1155/2018/5490491

**Published:** 2018-07-24

**Authors:** Jia Liu, Ziqiang Li, Hua Liu, Xianhua Wang, Chunxiao Lv, Ruihua Wang, Deqin Zhang, Yan Li, Xi Du, Yanfen Li, Baohe Wang, Yuhong Huang

**Affiliations:** ^1^Second Affiliated Hospital of Tianjin University of Traditional Chinese Medicine, Tianjin, China; ^2^Tianjin University of Traditional Chinese Medicine, Tianjin, China; ^3^Tianjin Medical University, Tianjin, China; ^4^Tianjin International Joint Academy of Biomedicine, Tianjin, China

## Abstract

Tangzhiqing tablet (TZQ) is derived from Tangzhiqing formula, which has been used to regulate glucose and lipid metabolism in China for hundreds of years. However, as a new Chinese patent medicine, its clinical indication is not clear. To explore the clinical indication and effect on the patients with type 2 diabetes mellitus (T2DM), a pilot clinical trial and metabolomics study were carried out. In the clinical study, T2DM patients were divided into three groups and treated with TZQ, placebo, or acarbose for 12 weeks, respectively. The metabolomic study based on UPLC Q-TOF MS was performed including patients with hypertriglyceridemia in TZQ and placebo groups and healthy volunteers. The clinical results showed that TZQ could reduce glycosylated hemoglobin (HbA1c) and fasting insulin. For patients with hypertriglyceridemia in TZQ group, the levels of HbA1c all decreased and were correlated with the baseline level of triglyceride. Metabonomics data showed a significant difference between patients and healthy volunteers, and 17 biomarkers were identified. After 12-week treatment with TZQ, 11 biomarkers decreased significantly (*p*<0.05), suggesting that TZQ could improve the metabolomic abnormalities in these participants. In conclusion, the clinical indication of TZQ was T2DM with hypertriglyceridemia, and its target was related to glycerophospholipid metabolism.

## 1. Introduction

Diabetes is a global health concern causing increasing morbidity, mortality, and social and economic burdens annually. According to the newly released Diabetes Atlas from the International Diabetes Federation (IDF), a total of 425 million adults had diabetes globally in 2017, and the number is expected to rise to 642 million by 2040 (www.diabetesatlas.org). During the past few years, the number of people with diabetes, especially type 2 diabetes mellitus (T2DM), is growing rapidly worldwide. Hypertriglyceridemia, which is tightly relevant to unhealthy lifestyle, is a common comorbidity in diabetes. Epidemiological studies in Asia and Europe found that hypertriglyceridemia is commonly associated with diabetes [1, 2]. Both of these two symbiotic disorders are recognized as independent risk factors for cardiovascular disease [3].

Traditional Chinese Medicine (TCM) has a long history in treating diabetes and hypertriglyceridemia, because of early intervention, multiple targets, and treatment based on syndrome differentiation. TCM can significantly retard diseases progression and improve quality of life. Tangzhiqing tablet (TZQ) is a Chinese patent medicine derived from Tangzhiqing formula which consists of five herbs,* Paeonia lactiflora* Pall., root,* Morus alba* L., leaf,* Nelumbo nucifera* Gaertn., leaf,* Salvia miltiorrhiza bge*., roots, and* Crataegus pinnatifida bge*., leaf [4]. The preclinical studies have shown that TZQ could significantly regulate the abnormal glucose and lipid levels in genetic type 2 diabetic KK-A^y^  mice [5–8] and high-carbohydrate/high-fat diet rats [9]. These effects are related to glucose and lipid absorption inhibition and free radical scavenging. The effects on glucose and lipid homeostasis are mediated through regulating adipocyte differentiation and insulin action by AMPK signaling pathway and PI3K/AKT signaling pathway [5–9]. Besides, TZQ can improve glucose metabolism by reducing *α*-glycosidase activity [5].

Metabolomics is used to evaluate the characteristics and interactions of low molecular weight metabolites under a specific set of conditions. It is a powerful analytical strategy to identify the metabolites* in vivo* and clarify metabolic pathways. Especially for metabolic disease, such as T2DM, metabolomics offers an opportunity to test multiple metabolic markers in large settings. Wang et al. found five branched-chain and aromatic amino acids had highly significant associations with future diabetes by the prospective Framingham Heart Study (FHS) using a 12-year follow-up [10]. Lu et al. found three branched-chain amino acids and four nonesterified fatty acids (NEFA) were potent predictors of diabetes development in Chinese adults [11]. Other metabolites, including phospholipids [11–14], acyl-carnitines [12, 13], amino acids [11–15], triglycerides [16], and small molecular weight compounds [11, 12, 14–16] have been defined as biomarkers for predicting T2DM, covering the glucose and phospholipid metabolism.

As a new Chinese patent medicine, the clinical indication of TZQ is still undefined, and the regulating effect on patients is not clear yet. In this study, we carried out a pilot clinical trial on T2DM patients to tentatively evaluate the clinical efficacy and determine clinical indication of TZQ. Moreover, the metabolomics strategy based on the ultraperformance liquid chromatography combined with quadrupole time-of-flight tandem mass spectrometry (UPLC Q-TOF MS) method combining with pattern recognition techniques was performed to investigate the changes in endogenous metabolites in serum between T2DM patients with hypertriglyceridemia and healthy volunteers.

## 2. Materials and Methods

### 2.1. Drugs and Reagents

TZQ (0.64 g), TZQ simulation agent (0.64 g), and acarbose simulation agent (50 mg) were obtained from Shandong Buchang Shenzhou Pharmaceutical Co., Ltd., which conducted Investigational New Drug Application (IND) study in November 2010 by CFDA (China Food and Drug Administration). Acarbose (50 mg) was produced by Bayer Healthcare Pharmaceuticals.

HPLC-grade acetonitrile was purchased from Merck (Merck, Germany). HPLC-grade formic acid was bought from ROE (ROE, USA). Deionized water was produced by Milli-Q ultrapure water system (Millipore, USA). Leucine-enkephalin was obtained from Sigma-Aldrich (MO, USA).

### 2.2. Study Subjects and Clinical Trial Design

The study subjects were recruited as part of a project entitled “Clinical trial to evaluate the efficacy and safety of TZQ on T2DM”. (The registration number from the international clinical trial net is ChiCTR-TTRCC-12002866.) All participants were adults in Tianjin region without renal or liver dysfunction. The study protocol was in accordance with the Helsinki declaration and approved by the Ethics Committee of the Second Affiliated Hospital of Tianjin University of Traditional Chinese Medicine. Written informed consent was obtained from all participants.

**Figure 1:**
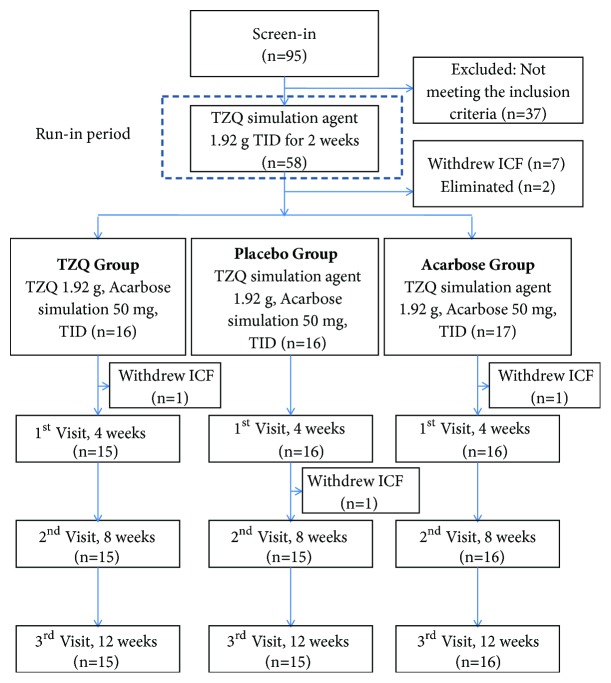
Flow diagram of the clinical trial.

Diabetic patients (with glycosylated hemoglobin (HbA1c) of 7.0%-9.0%) without any previous history of drug use and with ages between 18 and 75 were selected as subjects.  Figure 1 showed the flow diagram of this pilot clinical trial. The subjects who met the inclusion criteria were introduced to run-in period (TZQ simulation agent 1.92 g, TID) for 2 weeks and then entered the trial period for 12 weeks. The subjects were divided into three groups randomly: (1) TZQ group (TZQ 1.92 g, TID; acarbose simulation 50 mg, TID), (2) placebo group (TZQ simulation agent 1.92 g, TID; acarbose simulation 50 mg, TID), and (3) acarbose group (TZQ simulation agent 1.92 g, TID; acarbose 50 mg, TID). During the 12-week study period, a total of three visits (on the 4th, 8th, and 12th weeks) would be performed. During the process of the trial, the low fat diet was performed, including the fact that the calories from fat were no more than 30% of total calories, and the acceptable intake of saturated fatty acid was no more than 10%. The use of any other T2DM or hyperlipidemia treatment including oral medication, TCM therapy, and health care products was prohibited.

To investigate pharmacologic effects, the body mass index (BMI), waistline, glycosylated hemoglobin (HbA1c), fasting insulin (FINS), fasting blood glucose (FBG), oral glucose tolerance test at 2 h after oral ingestion of 75 g of glucose (OGTT2hBG), triglycerides (TG), total cholesterol (TC), low-density lipoprotein cholesterol (LDL-C), and high-density lipoproteins cholesterol (HDL-C) were measured at the beginning and the end of the study. To evaluate the safety of TZQ, routine clinical laboratory tests and electrocardiogram (ECG) were also measured.

### 2.3. Samples Collection and Preparation for Metabolomics

For metabolomics, each five subjects with hypertriglyceridemia (TG>1.70) in TZQ group and placebo group were selected. In addition, five healthy volunteers matched by age and sex were included in the normal group. They were screened by medical history, physical examination, and routine laboratory investigations. Their body mass index ranged between 19.67 and 21.84 kg/m^2^ (mean ± SD, 20.82±0.92 kg/m^2^). All of them signed the informed consent form. For TZQ group and placebo group, human blood samples were collected at the beginning and the end of the study. For normal group, blood samples were collected just once. Venous blood of every subject was collected in the morning before breakfast and was immediately centrifuged at 3500 rpm for 10 min and serum was transferred into a clean eppendorf tube. The serum samples were stored at -80°C until analysis.

Prior to analysis, the samples were thawed at room temperature and 100 *μ*L of serum samples was mixed with 300 *μ*L of acetonitrile. After being vortexed for 1 min and incubated for 10 min at 4°C, the mixture was centrifuged for 10 min at 13,000 rpm. The supernatant was transferred to a new eppendorf tube, followed by evaporation to dryness in a speedvac concentrator. Subsequently, the residue was resuspended in 100 *μ*L mobile phase prior to instrument analysis. Quality-control (QC) samples were prepared by mixing equal amount of serum from each sample and the metabolites were extracted using the same procedures as the test samples. One QC was inserted regularly before and after running every five samples.

### 2.4. Instrument Analysis

The ultraperformance liquid chromatography combined with quadrupole time-of-flight tandem mass spectrometry (UPLC Q-TOF MS) analysis was performed on Nexera X2 system (Shimadzu, Japan) coupled with a TripleTOF 5600 quadrupole-time-of-flight mass spectrometer (SCIEX, USA). Samples were separated on an Agilent ZORBAX Eclipse Plus C18 column (2.1×100 mm, 1.8 *μ*m). The column temperature was maintained at 40°C, and the flow rate was 0.25 mL/min. The mobile phase consisted of water (A) and acetonitrile (B) both containing 0.1% (v/v) formic acid. The gradient was initiated with 2% B for 1 min and then linearly increased to 90% B within 13 min. The gradient was kept at 90% B for 2 min and then back to 2% B within 0.1 min and held at 2 % B for another 4 min. The total run was 20 min. The Q-TOF mass spectrometer was run in positive mode. The data of m/z were collected for each test sample from 50 Da to 1500 Da. Parameters of MS were set as follows: capillary voltage: 3.0 kV; curtain gas: 35 psi; declustering potential: 100 V; collision energy: 10 V; interface heater temperature: 550°C.

### 2.5. Metabolomics Data Analysis

The raw data acquired by UPLC Q-TOF MS were imported to MarkerView software (version 1.2.1, SCIEX, USA) to conduct data pretreatment procedures, such as retention time alignment, peak discrimination, filtering, alignment, matching, and identification. After the algorithm operation of the software, a peak table with retention time (t_R_), m/z value, and corresponding peak intensity was generated. MetaboAnalyst 3.0 (http://www.metaboanalyst.ca/) was used for chemometrics analysis, such as principal components analysis (PCA) and partial least squares discriminate analysis (PLS-DA). To identify potential biomarkers, the accurate mass, retention time, and the fragmentation pattern from MS/MS had to be used. Besides, Human Metabolome Database (http://www.hmdb.ca/) was used to screen the potential biomarkers through m/z acquired by UPLC Q-TOF MS with molecular weight tolerance ±10 ppm. Open database sources, including MetaboAnalyst 3.0 (http://www.metaboanalyst.ca/) and KEGG (http://www.kegg.jp/), were used to identify the metabolic pathways.

### 2.6. Statistical Analysis

For clinical trial, all data were presented as mean ± SD and performed using SPSS 20.0 software. Paired t-test was used to assess the effects of each subject before and after the treatment. One-way analysis of variance (ANOVA) and covariance analysis were carried out to analyze the differences among the groups. Independent sample t-test was used to compare the baseline characteristics between case group and normal group. For metabolomics data, the mass and charge features of metabolites were log-transformed and auto scaled before PCA and PLS-DA. Besides, the univariate analysis, including fold change (FC) analysis and Student's* t*-tests, was also performed in MetaboAnalyst 3.0. Paired t-test was used to compare the changes of the peak intensity of the potential biomarkers before and after treatment in TZQ group and placebo group. All tests were two-tailed, and the level of significance was set at 0.05.

## 3. Results

### 3.1. Comparison of Baseline Characteristics among Three Groups

A total of 95 subjects were recruited for the study, and 58 subjects were suitable for inclusion criteria mentioned above. Except for 10 who withdrew due to personal reasons and 2 eliminated because of unstable (changes >15%) FBG or TG levels observed during the run-in period, 46 subjects completed the clinical trial, aged from 39 to 75. There were no adverse events in this study. The comparison of baseline characteristics among three groups was summarized in Table 1. The baseline characteristics of gender, age, BMI, waistline, HbA1c, FINs, FBG, OGTT2hBG, and blood lipids showed no statistically significant difference among three groups by ANOVA (*p* > 0.05).

### 3.2. Comparisons of Clinical Characteristics among Different Groups before and after Treatment

**Figure 2:**
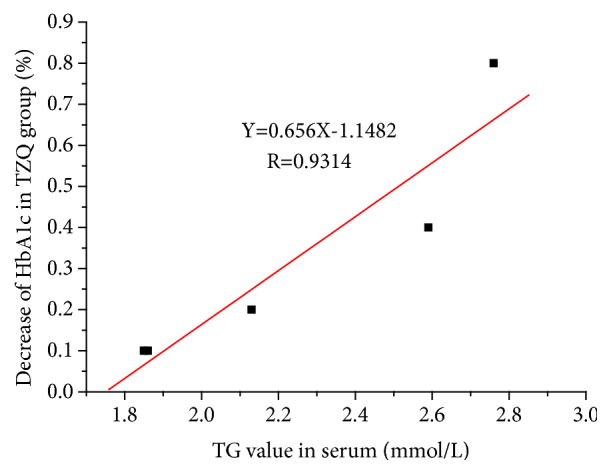
The relationship between the decrease of HbA1c and the corresponding TG baseline value (TG>1.7 mmol/L) in TZQ group (n=5).

After the 12-week treatment, a significant decrease of OGTT2hBG (*p*=0.020) and TG (*p*=0.044) from baseline was observed in acarbose group using paired t-test. Compared with placebo group, FINS (*p*=0.019) and OGTT2hBG (*p*=0.041) decreased significantly in acarbose group using covariance analysis. The decrease of HbA1c, FINs, and OGTT2hBG from baseline in TZQ group, although not statistically significant, was also observed (Table 2). For HbA1c, a decreasing tendency was observed in TZQ group (n=5) as TG>1.70 mmol/L (n=5). Interestingly, the decrease of HbA1c was positively correlated with the baseline level of TG (Figure 2).

### 3.3. Clinical Subjects in Metabolomics

To demonstrate that TZQ had more dramatic hypoglycemic effect on T2DM patients with hypertriglyceridemia, each five subjects with TG>1.70 mmol/L from TZQ group and placebo group were selected to metabolomics (case group). In addition, five healthy volunteers matched by age and sex were admitted into normal group. Demographic and clinical parameters of these groups were shown in Table 3.

### 3.4. The Detection Analysis of the Metabolomics by UPLC Q-TOF MS

UPLC Q-TOF MS analysis was carried out to acquire metabolic profiles. Operation conditions were optimized to elute as many metabolites as possible in a single injection. Finally, a total of 4859 positive ion mode features were extracted from the UPLC Q-TOF MS data. QC data showed that the RSD of retention time and peak intensity of m/z 132.1 (1.94 min), m/z 302.3 (8.48 min), and m/z 496.3 (4.16 min) were 0.21%, 0.47%, 0.85%, and 9.76%, 8.64%, 8.98%, respectively. The results indicated the good stability and reproducibility during the whole analysis procedure.

### 3.5. Metabolomics and Pathway Analysis

**Figure 3:**
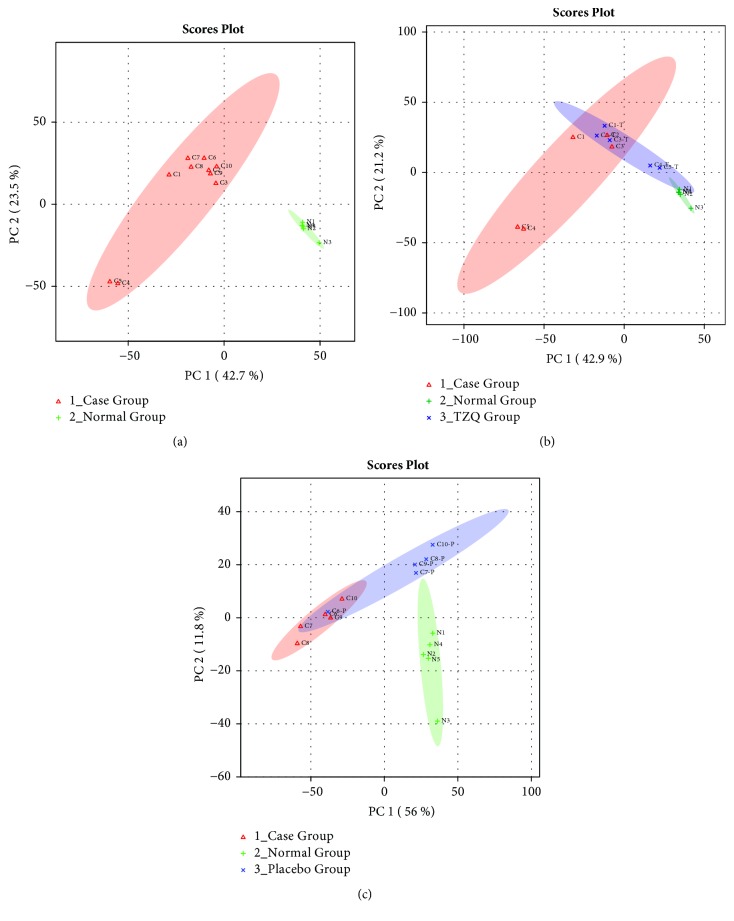
Principal components analysis (PCA) scores plot of case group and normal group (a); case group, normal group, and TZQ group (b); and case group, normal group, and placebo group (c).

To distinguish case group and normal group based on UPLC Q-TOF MS spectra and understand their endogenous metabolic differences, principal components analysis (PCA) was carried out. Case group and normal group contained 10 and 5 samples, respectively. The results of PCA scores plot showed that the two groups were separated with no overlap (Figure 3(a)), indicating that the serum metabolic pattern was significantly different between the case group and normal group.

The variable importance in the projection (VIP) in partial least square discriminate analysis (PLS-DA) and t-test analysis identified metabolites that were significantly different between case group and normal group. The potential biomarkers were identified by the fragmentation pattern from MS/MS and ions with VIP>1 and* p*<0.05.  Table 4 showed the potential biomarkers and possible metabolite pathways. The levels of diacylglycerol (DG(22:0/20:5), DG(15:0/18:3)), phosphatidylcholine (PC(15:0/20:2), PC(16:0/22:4), PC(14:0/14:1)), phosphatidylethanolamine (PE(14:0/18:4), PE(14:0/20:4)), lysophosphatidylcholine (LysoPC(16:0), LysoPC(18:0), LysoPC(18:3), LysoPC(14:1)), D-galactose, uridine diphosphate glucose (UDP-glucose), L-leucine, and L-tyrosine in patients were significantly higher than that in healthy volunteers, whereas sphinganine and cholest-5-ene were significantly lower.

To determine whether TZQ affected the metabolic pattern of patients and to find the metabolites with significant changes, PCA was used. The results of scores plot showed that the case group and normal group were separated clearly, and the five cases with TZQ treated for 12 weeks (Figure 3(b)) were mainly located between the case group and the normal group. While the other five cases with placebo treated (Figure 3(c)) were much closer to the case group. Combined with the result of clinical effect, this change of serum metabolic pattern showed that the patients were exhibiting a tendency to recovering to normal group after TZQ treatment, which agreed with the change of HbA1c.  Table 5 indicated that LysoPC(16:0), LysoPC(18:3), LysoPC(14:1), PC(15:0/20:2), PC(16:0/22:4), PC(14:0/14:1), PE(14:0/20:4), DG(22:0/20:5), and DG(15:0/18:3) decreased significantly (*p*<0.05) after the treatment of TZQ. These results indicated that some metabolites had tendency to come back from the case group to the normal group after TZQ treatment.

**Figure 4:**
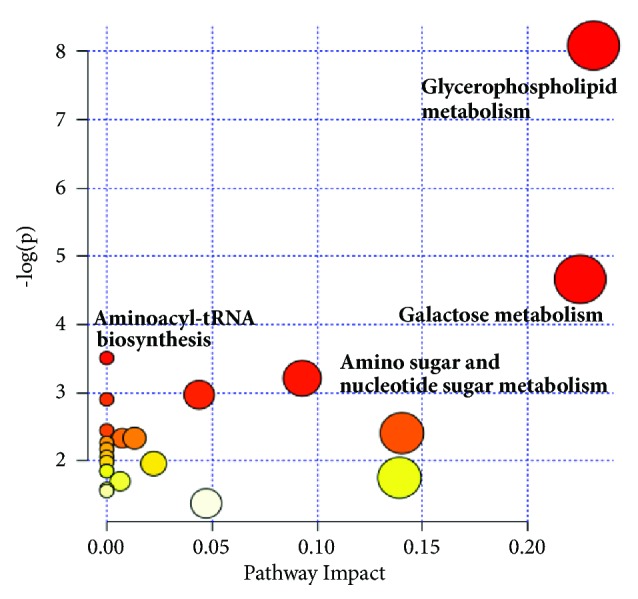
The main metabolic pathways based on potential biomarkers.

On the basis of 17 potential biomarkers between the case group and normal group, the pathway analysis based on MetaboAnalyst 3.0 was carried out. As shown in Figure 4, the main pathways between T2DM patients with hypertriglyceridemia and healthy volunteers were glycerophospholipid metabolism, galactose metabolism, aminoacyl-tRNA biosynthesis, and amino sugar and nucleotide sugar metabolism (*p*<0.05). Compared with the peak intensity in the different groups, TZQ treatment drove glycerophospholipid metabolism in patients to return to normal gradually.

## 4. Discussion

In this study, a pilot clinical trial on T2DM patients for evaluating the clinical efficacy and determining clinical indication of TZQ was carried out. The results showed that TZQ was beneficial for T2DM with hypertriglyceridemia. Metabolomics revealed that TZQ regulated the disorder of glycerophospholipid metabolism in T2DM patients with hypertriglyceridemia.

### 4.1. Clinical Trials

Patients with TZQ treatment (1.92g, TID) for 12 weeks had a trend to lower the values of FINS, OGTT2h, HbA1c, waistline, and BMI based on this pilot clinical trial. Compared with other diagnostic criteria for diabetes, HbA1c level is correlated with blood glucose level and can represent the average blood glucose level within 4 to 12 weeks. While FBG, OGTT2hBG, and random blood glucose can only represent transient blood glucose and be impacted by nervousness. This trial indicated that TZQ could decrease the level of HbA1c for the patients with TG>1.70. Moreover, the decrease of HbA1c was positive correlation with the baseline level of TG. The results of HbA1c demonstrated that TZQ treatment for 12 weeks continuously had an effective hypoglycemic activity on T2DM patients with hypertriglyceridemia. Besides, the sample size could be estimated through this pilot clinical trial as follows [17, 18]:

(1) m=Z1−α/2+Z1−β22σ2δ2

(2) σ2=n1−1S12+n2−1S22n1+n2−2

where m is the sample size required in each group; *α* is the significance level; Z_1-*α*/2_ is the 100(1-*α*/2)th percentile of the standard normal distribution and depends on level of significance, Z_1-*α*/2_=1.960 for *α*=0.05; 1-*β* is the power; Z_1-*β*_ is the 100(1-*β*)th percentile of the standard normal distribution and depends on power, Z_1-*β*_=0.845 for 1-*β*=0.8; n is the number of every group of the pilot clinical trial; S^2^ is the variance in the change of outcome in every group; *δ* is the margin based upon a combination of statistical reasoning and clinical importance, generally ranged from 1/5 to 1/2 of *σ*^2^, here, *δ*=0.4 for both noninferiority trial and superiority trial.

According to the equations above, a significant difference (*p*<0.05) would be detected for the primary outcome HbA1c between TZQ group and acarbose group (noninferiority trial) or between TZQ group and placebo group (superiority trial) if the sample size increased to 92 or 108.

### 4.2. Metabolomics Study

**Figure 5:**
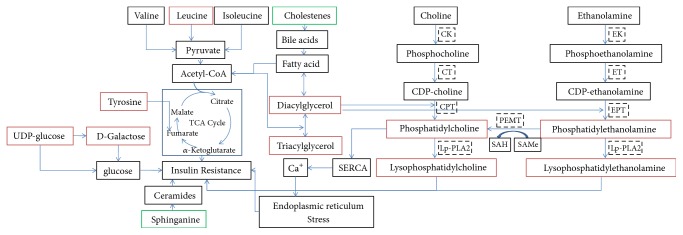
The relationship between insulin resistance and 17 potential biomarkers. Red represented the biomarkers whose peak intensity rose in case group, and green represented the biomarkers whose peak intensity reduced in case group (CK, choline kinase; CT, CTP phosphocholine cytidylyltransferase; CPT, CDP-choline:1,2-diacylglycerol cholinephos-photransferase; lp-PLA2: phospholipase A2; EK, Ethanolamine kinase; ET, phosphoethanolamine cytidylyltransferase; EPT, CDP-ethanolamine:1,2-diacylglycerol ethanolaminephosphotransferase; PEMT, phosphatidylethanolamine N-methyltransferase; SAH, S-adenosylhomocysteine; SAMe, S-adenosylmethionine).

Compared with healthy volunteers, 17 potential biomarkers were identified as risk of T2DM, including increased levels of D-galactose, UDP-glucose, L-leucine, L-tyrosine, LysoPC species, PC species, PE species, and DG species, and decreased levels of sphinganine and cholest-5-ene. These potential biomarkers refer to glucose, amino acid, and lipid metabolism, and the relationship to insulin resistance was illuminated in Figure 5.

After the 12-week treatment with TZQ, a serial of PC, PE, lysoPC, and DG was decreased, while other metabolites were not changed significantly. Briefly, TZQ influenced the glycerophospholipid metabolism directly and regulated the glucose and lipid metabolism to treat T2DM with hypertriglyceridemia.

### 4.3. Glycerophospholipid Metabolism

In this study, TZQ had strong impact on glycerophospholipids, including LysoPC, PC, PE, and DG, which were significantly elevated in case group (*VIP*>1 and* p*<0.05). PC and PE are the largest amount of glycerophospholipid in mammals. They are classes of important constituents in the biomembranes and provide the majority of membrane lipids within cells. In most types of cells, the synthesis of PC and PE is both through DG, which is related to insulin resistance [19]. Glycerophospholipid species, which are synthesized downstream of DG, are probable contributors to metabolic diseases. In fact, the increase of PC and PE was related strongly to obesity, diabetes, and other metabolic syndromes [20, 21].

PC plays an important role in the lipid storage to form lipid droplets and lipoproteins, which are both increased in obesity. Some researchers indicated that the increase of PC to liver-derived microsomes* in vitro* or the additional PC content in the membrane inhibited the calcium transport activity of sarcoendoplasmic reticulum Ca^2+^-ATPase (SERCA), which maintained calcium homeostasis in this organelle principally [22, 23]. This contributed to protein misfolding and endoplasmic reticulum (ER) stress and resulted in insulin resistance finally [24]. Selathurai et al. verified that phospholipids were the probable modulators of muscle insulin resistance rather than diacylglycerol or triacylglycerol, through muscle-specific knocking out of ethanolamine-phosphate cytidylyltransferase in mice, which retained insulin sensitivity and showed marked increases in mitochondrial biogenesis and muscle oxidative capacity compared with wild-type mice [25].

LysoPC belongs to the class of glycerophospholipids and is generated by PC hydrolysis through phospholipase catalyzing. Because of the high activity of phospholipase in diabetes, the hydrolysis of the sn-2 position of glycerophospholipids increased. Therefore, LysoPC in patients is much more than normal. Han et al. used various pharmacological inhibitors to find evidence favoring the role of LysoPC produced from FFA in insulin resistance* in vitro* and* in vivo* [26].They implicated LysoPC as an important lipid intermediate that links saturated fatty acids to insulin resistance.

### 4.4. Other Metabolisms

In this study, the differences between the case group and normal group in carbohydrate metabolism, amino acid metabolism, and other lipid metabolism were also found. These metabolisms were implicated in T2DM, but they were not the main targets for TZQ. This could be the reason that TZQ had no influence on patients with isolated disorder of glucose metabolism.

The level of D-galactose and UDP-glucose was higher in patients and was significantly associated with T2DM. D-Galactose is an aldohexose, which occurs naturally in the D-form in lactose, cerebrosides, gangliosides, and mucoproteins. Galactose is an isomeride of glucose and can be rapidly converted to glucose through the Leloir pathway [27]. Similar to glucose, D-galactose is an energy-providing metabolite and also a necessary basic substrate for the biosynthesis of many macromolecules in the body. Increased galactose metabolism may lead to long-term, gradual increases in serum glucose and may result in insulin resistance. UDP-glucose is a key intermediate in carbohydrate metabolism. Served as a precursor of glycogen, UDP-glucose can be metabolized into UDP-galactose and UDP-glucuronic acid, which can be incorporated into polysaccharides as galactose and glucuronic acid. UDP-glucose also serves as a precursor of sucrose lipopolysaccharides and glycosphingolipids[28].

L-leucine belongs to branched-chain amino acid (BCAAs) along with isoleucine and valine, which play an essential role in the insulin secretion, regulation of protein turnover, and protein synthesis [12]. The additional fasting concentrations of circulating BCAAs are associated with an elevated risk of T2DM, insulin resistance, and other metabolic diseases in humans because of the decreased activity of branched-chain *α*-keto acid dehydrogenase [29]. L-tyrosine, as one part of aromatic amino acid (AAAs), is a glucogenic and ketogenic amino acid. Some studies revealed that L-tyrosine has positive effects on the development of diabetes, as well as BCAAs [30]. In 2011, the Framingham Offspring Study reported that increased BCAAs (isoleucine, leucine, and valine) and AAAs (tyrosine and phenylalanine) were able to predict the risk of diabetes up to 12 years prior to disease onset [10].

Sphinganine belongs to sphingolipids and is a key precursor on ceramide de novo synthesis pathway [31]. Several studies have suggested that ceramides and other sphingolipids in cells play a direct role in reducing insulin sensitivity [32]. However, sphinganine in serum of patients was lower than healthy volunteers in this study. Cholestenes are derivatives of cholestanes which have a double bond. They are principally responsible for the synthetic process of bile acids. Some studies showed that the activation of bile acid–responsive G protein–coupled receptor TGR5 may promote pathways that were protective against diet-induced diabetes [33]. Therefore, the reduction of cholestenes would be related to the development of T2DM.

## 5. Conclusions

In this study, the clinical trial indicated that TZQ was appropriate for T2DM patients with hypertriglyceridemia. The decrease of HbA1c was positive correlation with the baseline level of TG. Metabolomics study based on UPLC Q-TOF MS and chemometrics analysis illustrated that TZQ influenced glycerophospholipid metabolism to regulate the disorder of glucose and lipid metabolism.

This was a pilot clinical trial to explore the clinical efficacy and effect on endogenous metabolites for T2DM of TZQ because of the small sample size. If we enlarge the sample size and eliminate the difference of genetic background, with uniform diets and living habits, the results will be more meaningful. Based on this study, a multicenter clinical trial will be carried out by our team to evaluate the safety and efficacy of TZQ on the T2DM with hypertriglyceridemia in the near future.

## Figures and Tables

**Table 1:** The comparison of baseline characteristics among three groups (mean ± S.D.).

Variable	TZQ-F Group	Placebo Group	Acarbose Group
	(n=15)	(n=15)	(n=16)
Sex (M/F)	8/7	12/3	9/7
Age (year)	62.93±6.11	57.40±9.74	58.07±5.23
BMI (kg/m2)	26.99±3.17	28.56±3.87	25.90±2.27
Waistline (cm)	95.43±11.24	100.23±8.36	94.87±8.44
HbA1c (%)	7.64±0.81	7.59±1.03	7.25±0.79
FINS (mIU/L)	16.64±8.65	15.00±7.07	15.10±14.57
FBG (mmol/L)	7.44±1.17	7.75±1.07	7.62±1.17
OGTT2hBG (mmol/L)	13.96±4.02	13.76±3.15	13.91±4.68
TG (mmol/L)	1.52±0.61	2.06±1.05	2.35±1.38
TC (mmol/L)	4.92±0.70	4.87±0.82	4.74±0.78
LDL-C (mmol/L)	2.86±0.52	2.87±0.61	2.59±0.63
HDL-C (mmol/L)	1.38±0.39	1.17±0.25	1.22±0.35

Note: data were analyzed using ANOVA. *p*>0.05 (among three groups). FBG: fasting blood glucose; OGTT 2hBG: oral glucose tolerance test 2 h blood glucose; HbA1c: glycosylated hemoglobin; BMI: body mass index; TC: total cholesterol; TG: triglyceride; HDL: high-density lipoprotein cholesterol; LDL: low-density lipoprotein cholesterol.

**Table 2:** The changes comparison of clinical characteristics before and after treatment in three groups (mean ± S.D.).

Variable	TZQ Group (n=15)		Placebo Group (n=15)		Acarbose Group (n=16)	
	Before treatment	After treatment	Before treatment	After treatment	Before treatment	After treatment
BMI (kg/m2)	26.99±3.17	25.41±3.44	28.56±3.87	28.53±3.44	25.90±2.27	25.69±2.33
Waistline (cm)	95.43±11.24	92.77±10.29	100.23±8.36	100.60±8.29	94.87±8.44	93.63±7.74
HbA1c (%)	7.64±0.81	7.52±1.18	7.59±1.03	7.67±0.74	7.25±0.79	7.16±0.92
FINS (mIU/L)	16.64±8.65	14.28±8.29	15.00±7.07	15.73±7.77	15.10±14.57	10.42±4.97_ _^△^
FBG (mmol/L)	7.44±1.17	7.85±2.11	7.75±1.07	7.98±1.43	7.62±1.17	7.27±1.22
OGTT2hBG (mmol/L)	13.96±4.02	13.72±4.89	13.76±3.15	13.90±4.65	13.91±4.68	12.03±4.30_ _^*∗*△^
TG (mmol/L)	1.52±0.61	1.88±1.51	2.06±1.05	2.17±1.24	2.35±1.38	1.81±1.18_ _^*∗*^
TC (mmol/L)	4.92±0.70	4.99±0.93	4.87±0.82	4.95±1.05	4.74±0.78	4.56±0.90
LDL (mmol/L)	2.86±0.52	2.91±0.70	2.87±0.61	2.85±0.79	2.59±0.63	2.52±0.73
HDL (mmol/L)	1.38±0.39	1.30±0.40	1.17±0.25	1.17±0.30	1.22±0.35	1.24±0.40

Note: data were analyzed using paired t-test or covariance analysis. ^*∗*^*p*< 0.05 (compared with the same group before treatment, paired t-test), ^△^*p* < 0.05 (compared with placebo group, covariance analysis). FBG: fasting blood glucose; OGTT 2hBG: oral glucose tolerance test 2 h blood glucose; HbA1c: glycosylated hemoglobin; BMI: body mass index; TC: total cholesterol; TG: triglyceride; HDL: high-density lipoprotein cholesterol; LDL: low-density lipoprotein cholesterol.

**Table 3:** The comparison of baseline characteristics between case group and normal group in metabolomics (mean ± SD).

Variable	Normal group (n=5)	Case group (n=10)			
		TZQ group (n=5)		placebo group (n=5)	
	mean ± SD	mean ± SD	*p*	mean±SD	*p*
Sex (M/F)	2/3	2/3		4/1	
Age (year)	60.60±1.95	62.60±6.43	0.524	49.80±8.76	0.027
BMI (kg/m2)	20.82±0.92	24.92±3.80	0.047	29.32±4.37	0.003
Waistline (cm)	73.60±4.72	93.30±16.10	0.031	98.40±7.27	<0.001
HbA1c (%)	4.66±0.92	7.84±0.75	<0.001	7.96±0.71	<0.001
FINS (mIU/L)	9.50±4.29	16.71±8.87	0.140	14.66±5.90	0.153
FBG (mmol/L)	4.66±0.50	6.89±1.30	0.007	8.29±1.34	<0.001
OGTT2hBG (mmol/L)	9.26±0.92	12.38±1.32	0.002	14.42±1.46	<0.001
TG (mmol/L)	1.04±0.06	2.24±0.42	<0.001	2.61±0.47	<0.001
TC (mmol/L)	3.47±0.68	5.29±0.64	0.002	4.88±0.39	0.004
LDL-C (mmol/L)	1.88±0.61	3.06±0.41	0.007	2.88±0.36	0.013
HDL-C (mmol/L)	1.50±0.22	1.21±0.26	0.087	0.95±0.28	0.009

Note: data were analyzed using independent sample t test. FBG: fasting blood glucose; OGTT2hBG: oral glucose tolerance test 2 h blood glucose; HbA1c: glycosylated hemoglobin; BMI: body mass index; TC: total cholesterol; TG: triglyceride; HDL: high-density lipoprotein cholesterol; LDL: low-density lipoprotein cholesterol.

**Table 4:** The potential biomarkers and possible metabolite pathways.

m/z	t_R_	Metabolite	Class	HMDB	VIP	*p* value	Trend^*∗*^	Metabolic pathways
181.1559	1.52	D-Galactose	Carbohydrate	00143	1.5497	<0.001	up	Galactose metabolism
567.3018	2.13	UDP-glucose		00286	1.3043	0.002	up	
132.1018	1.94	L-Leucine	amino acid	00687	1.4244	<0.001	up	Aminoacyl-tRNA biosynthesis
182.0809	1.85	L-Tyrosine		00158	1.1778	0.002	up	
496.3386	4.16	LysoPC(16:0)	Glycerophospholipid	10382	1.4823	<0.001	up	Glycerophospholipid metabolism
524.3685	5.15	LysoPC(18:0)		10384	1.0618	0.008	up	
518.3238	4.79	LysoPC(18:3)		10387	1.5689	0.007	up	
466.2898	3.83	LysoPC(14:1)		10380	1.3510	<0.001	up	
772.5782	8.97	PC(15:0/20:2)		07946	1.1258	0.004	up	
810.6019	9.38	PC(16:0/22:4)		07988	1.0647	0.008	up	
676.4853	8.26	PC(14:0/14:1)		07867	1.5373	<0.001	up	
684.4608	9.54	PE(14:0/18:4)		08832	1.4224	<0.001	up	
712.4935	9.99	PE(14:0/20:4)		08838	1.5114	<0.001	up	
699.5945	11.93	DG(22:0/20:5)	glyceride	07607	1.4373	<0.001	up	
577.4806	11.03	DG(15:0/18:3)		07076	1.005	0.014	up	
302.3058	8.48	Sphinganine	Sphingolipid	00269	1.4622	<0.001	down	Sphingolipid metabolism
371.3699	9.81	Cholest-5-ene	Cholestene	00941	1.3602	<0.001	down	- - -

Note: Data were analyzed by Student's *t*-tests. ^*∗*^Trend: Case group versus Normal group.

**Table 5:** The changes comparison of the peak intensity of the potential biomarkers before and after treatment in two groups (mean ± S.D.).

Biomarkers	TZQ Group (n=15)			Placebo Group (n=15)		
	Before treatment	After treatment	*p*	Before treatment	After treatment	*p*
D-Galactose	2097.23±555.64	1796.80±1050.53	0.610	2217.10±464.44	2486.60±1038.89	0.579
UDP-glucose	1220.75±685.75	468.26±308.37	0.155	682.36±217.58	882.12±213.60	0.086
L-Leucine	3490.38±274.78	3397.90±76.80	0.470	3505.53±239.78	4093.26±1029.48	0.331
L-Tyrosine	1667.88±208.55	1573.02±135.06	0.276	1579.23±185.80	1847.48±480.83	0.237
LysoPC(16:0)	9833.36±1318.86	3633.52±1197.10	<0.001	6021.73±1873.12	5961.09±4533.85	0.969
LysoPC(18:0)	751.23±118.43	742.64±381.31	0.958	863.39±81.89	850.71±315.53	0.926
LysoPC(18:3)	18365.49±1468.24	8804.01±1598.84	<0.001	16399.05±1399.12	15440.55±4240.13	0.513
LysoPC(14:1)	626.84±296.06	157.11±18.67	0.026	517.33±293.07	299.37±381.13	0.294
PC(15:0/20:2)	781.42±142.81	489.05±169.67	0.001	840.39±64.15	893.06±417.55	0.778
PC(16:0/22:4)	7471.85±2200.75	3462.13±407.02	0.015	6396.02±2356.44	4846.48±1015.19	0.222
PC(14:0/14:1)	1081.27±366.46	186.24±47.03	0.005	1062.27±340.92	1141.66±292.78	0.665
PE(14:0/18:4)	816.29±661.96	226.31±196.64	0.198	360.05±91.35	511.65±125.01	0.124
PE(14:0/20:4)	1659.73±611.85	642.14±142.21	0.026	1867.68±441.45	1896.16±1102.87	0.966
DG(22:0/20:5)	1171.63±515.18	365.11±60.69	0.017	935.65±219.09	916.33±549.78	0.952
DG(15:0/18:3)	1549.78±708.90	415.63±292.41	0.007	779.78±508.12	835.61±482.38	0.860
Sphinganine	3594.59±5522.04	1203.80±287.44	0.396	1302.85±151.44	1205.19±355.10	0.451
Cholest-5-ene	28.69±29.76	37.82±34.07	0.341	7.89±4.14	17.73±18.12	0.326

Note: data were analyzed using paired t-test.

## Data Availability

The data used to support the findings of this study are available from the corresponding author upon request.
